# Impact of community care services on the health of older adults: evidence from China

**DOI:** 10.3389/fpubh.2023.1160151

**Published:** 2023-04-18

**Authors:** Wenjing Ma, Zheng Shen

**Affiliations:** ^1^School of Economics and Trade, Henan University of Animal Husbandry and Economy, Zhengzhou, China; ^2^School of Economics and Management, Zhejiang A&F University, Hangzhou, China

**Keywords:** community care services, health promotion, aging, China, health of older adults

## Abstract

**Introduction:**

The rapid growth in the population of older adults has put tremendous pressure on medical and social services in countries including China. Community care services are a feasible solution for promoting healthy aging in developing countries. This study investigated the association between community care services and the health of older adults in China.

**Method:**

Using nationally representative survey data from China, consisting of four waves conducted in 2005, 2008, 2011, and 2014, a balanced panel dataset was constructed using a sample of 4,700 older adults (33.1% aged 80 years or older, 51.0% residing in rural areas, and 48.8% women). We employed linear regression models with time-fixed effects and instrumental variable approaches to estimate the effect of community care services on the health of older adults, as well as the differences in these effects across subgroups.

**Results:**

The results showed that community care services lead to a significant improvement in both the objective and subjective health and wellbeing of older adults. Among the various service offerings, spiritual recreation services led to a significant increase in both objective and subjective health scores, while medical care services significantly improved wellbeing. This suggests a varied effect of subdivided service types. Further evidence suggests that spiritual recreation services have a significant health-enhancing effect on multiple groups of older adults, and the effect of medical care services is more effective for those living in rural areas, women, and those who are older than 80 years (all *p* < 0.05).

**Discussion:**

Few studies have examined the impact of community care services on the health of older adults in developing countries. The findings present important implications for improving the health status of older adults and provide suggestions for establishing a socialized aged care system in China.

## 1. Introduction

Organizing care for older adults is a serious challenge in the face of global population aging. Institutional care is not widely available and is unlikely to become widespread because of its high costs in many underdeveloped countries (1, 2). Considering the growing burden of providing care for older adults, many countries have opted to promote independent living in the community (3, 4).

Since China became an aging society in 2000, the population aged 65 and older has reached 200.56 million (14.2% of the total population), while the number of beds in residential long-term care facilities is only 8.135 million.^1^ The rapid growth in the population of older adults has put tremendous pressure on medical and social services. Given the small size of family structures, the traditional family-based elderly care system is no longer sustainable in China (5–8). Similar to other developing countries that are growing old before becoming rich, China is unable to provide sufficient institutional nursing beds for its large elderly population. In this condition, prioritizing family-based community care services is necessary to meet the health needs of the elderly (2, 9), improve their health, and extend their life expectancy (10).

However, currently, there is not enough evidence to determine the impact of community care services on the health of the elderly in developing countries. It is also unclear which types of services are more effective in improving the health of the elderly. This lack of empirical evidence makes it difficult to formulate effective social care policies and determine which services should be given priority with limited resources. Therefore, it is important to evaluate the impact of community care services on the health of the elderly, especially through the examination of different types of services.

## 2. Literature review

The health of older adults not only refers to their physical health but also more importantly their life satisfaction (11). This is because although the decline of physical organs and physiological functions with age is inevitable, older adults can achieve “healthy aging” through better psychological health. Among many variables that affect the health of older adults, scholars have begun to pay special attention to the old-age care model. One reason is that this factor is important for improving the health of older adults, and the other is that policymakers need to assess the impact of different old-age care services to promote their successful development. Several studies have found a significant relationship between the old-age care model and the health of older adults (12, 13). It is evident that mortality was significantly lower among older adults living with others compared to those living alone (14), and aging in places with economic and residential independence significantly affected the health and subjective wellbeing of older adults (15). It is difficult for older adults to decide whether to move from their home to an alternative care location (16, 17). Achieving aging in place is related to the availability of formal or informal support, including material security, emotional support, and timely treatment (18). This suggests that the key determinants of older adults' healthcare are access to life care, medical support, and spiritual support provided by families or socialized organizations.

The World Health Organization's Action Plan on Aging and Health highlights the importance of delivering home- and community-based care to enable older adults to “age in place” with dignity (19). Community care services allow older adults to age in place while providing support such as life care, medical care, and spiritual recreation (20, 21). Some studies point to the positive health impacts of community care services in developing countries, which are designed to meet basic physical- and higher-level spiritual needs (22–24). Different services have different goals and roles in achieving healthy aging (25). One is to prevent the decline of the physical health of older adults, through things like medical care services, which are key in supporting healthy aging (26, 27). Another is to improve the psychological state of loneliness of older adults by keeping them connected with the community. One such example is the provision of life care services that relieve the stress of family caregivers, which benefit older adults' families and thus contribute to life satisfaction (28, 29).

There is insufficient evidence to determine the impact of community care services on the health of older adults in developing countries. Since China has been carrying out community care services for a relatively short period of time, two main theories related to community home care services and the health of older adults have been formed by the academic community. First, from a positive perspective, community home-based care services are conducive to improving physical health and reducing the risk of chronic diseases (30, 31). Other scholars believe that the life satisfaction and depressive symptoms of older adults in the home-based community care model are better than those in institutional care (32, 33). Second, from the problem perspective, the home-based community care model can provide family and social support, but in practice, it does not achieve the effect of 1 + 1>2 (34). Community services have failed to coordinate with home care due to the unregulated nature of community care services, absence of diverse services, long distances from home, and insufficient quantity. If the external stimulation received by older adults living at home is weakened, this may increase their loneliness and isolation (9).

Existing studies on the relationship between community care services and health in China focus on one aspect of health, treat community care services as a whole ignore the differences in the types within them, or present endogenous issues. This study focuses on answering the following four questions: What is the impact of community care services on the health of older adults in China? What type of service has the greatest impact? How do the effects of these impacts differ among older adults of different ages, residences, and genders? How can policies provide targeted community care services?

This study extends existing research on “community care services and health” in three ways. First, as the existing literature on the relationship between community care services and health often suffers from endogeneity, we developed linear regression models (controlling for time periods and regional fixed effects) and reliable instrumental variables (IV) for the independent variables using an IV model to reduce endogeneity bias and obtained robust and credible estimation results. Second, we extended the connotation of older adults' health in multidimensional terms, using three indicators to represent the health of older adults: objective health, subjective self-rated health, and subjective wellbeing, which is closer to real-life health evaluation. Third, recognizing that the relationship between community care services and older adults' health may be affected by service content, we subdivided the community care services into four types: life care, healthcare, spiritual recreation, and legal advocacy services.

The main findings of this study are as follows. First, community care services had a generalized effect on the health and wellbeing of the older adult. Second, spiritual recreation and medical care services had a positive effect on the health of the older adult. Third, medical care services had more obvious effects on the health of the rural population, women, and those that are older than 80 years; and spiritual recreation services had a general health-enhancing effect on the older adult. Fourth, the government's prioritization of spiritual recreation services had good health-enhancing effects and medical care services that are important and inadequate for rural older adults. We suggest that providing targeted and diversified services promote healthy aging.

## 3. Conceptual framework

The health demand theory (35) suggests that the initial stock of health capital is determined innately, and its depreciation rate will continue to increase with age. Therefore, the stock of health capital among consumers appears to decline; to avoid this, consumers can increase their investment in health capital by purchasing medical and other services. Thus, as consumers age, the health depreciation rate increases, and the demand for health services increases. The health production function proposed by Grossman (35), which treats health as a function of medical care, income level, health behavior, and socioeconomic status, constitutes the basis of the health demand model.

Scholars have extended the Grossman health production function to include health as a function of health services, income level, health behavior, socioeconomic status, and other factors (36–38). Liu et al. (15) expanded this by dividing health services into short-term medical care and long-term care services as inputs for health production. According to the community-based home care model, long-term care services are mainly provided by families and communities (4). This study uses Grossman (35) as a starting point and includes community care services as an input for health production:


(1) $$\begin{array}{l} H=f\left(CS,FS,MC\;,\;Y,H_{0},X\right), \end{array}$$

where *H* represents the health of the older adult, which is explained by the community care services (*CS*), family support (*FS*), income (*Y*), initial health status (*H*_0_), and other variables (*X*) that represent the socioeconomic characteristics of the older adult.

## 4. Materials and methods

### 4.1. Data

This study used data from the Chinese Longitudinal Healthy Longevity Survey (CLHLS), organized by Peking University, and publicly available data from the Peking University Open Research Data Platform. In 1998, the baseline survey randomly selected ~50% of counties and cities in 22 of China's 31 provinces, covering 85% of China's total population. The study targeted institutionalized and community-living older adults. All centenarians who agreed to participate in the selected areas were enrolled in the study. Additionally, octogenarians and non-agenarians were randomly selected based on gender and place of residence (i.e., living in the same street, village, city, or county) as the centenarians. This matching recruitment method resulted in an over-representation of the oldest old and older men in the baseline. The 1998 and 2000 surveys interviewed only the oldest old but the 2002, 2005, and 2008 surveys interviewed the elderly aged between 65 and 79 years and middle-aged persons between 35 and 64 years as well. In the subsequent survey years, samples were replenished in the areas where samples were lost due to death. In the CLHLS, a weight of age–sex–urban/rural residence in the sample with the distribution of the total population in the sampled 22 provinces was employed to reflect the unique sampling design. The data are highly representative, and a detailed description of the study population has been previously published (39, 40).

The survey mainly covered self-rated health status and quality of life status, mobility, residence patterns, economic sources, medical coverage, health behaviors, and demographic and sociological characteristics of the participants. At the same time, medical school students or local healthcare workers who served as investigators conducted basic health examinations of the older adults to provide the objective health status of the interviewed older adults. Participants were followed up in 2000, 2002, 2005, 2008, 2011, and 2014. The survey of the communities where the older adults lived was added in 2005, including the questionnaire on community care services. All the interviewers were intensively trained before the survey. All procedures involving human subjects/patients were approved by Peking University.

In this study, we selected CLHLS data from 2005, 2008, 2011, and 2014, and after excluding the sample of older adults who chose institutionalized care, aged under 80 years, incomplete health information, and incomplete information on community care services, a total of 4,700 participants from four waves (2005, 2008, 2011, and 2014) were selected to construct a balanced panel dataset. The mean age of the total sample was 77.255 (SD 7.355). There were 2,397 (51%) participants who lived in rural areas, and 2,303 (49%) participants who lived in urban areas. The number of male participants was 2,408 (51.2%), and the number of female participants was 2,292 (48.8%).

### 4.2. Outcome variables

The health of the older adults studied in this article includes both physical and psychological aspects, and the following three indicators are used as key dependent variables to measure the health of the older adults.

The first is objective health, a variable in which the investigator observes and records the health of the interviewee on a scale ranging between 0 = very bad, 1 = bad, 2 = relatively good, and 3 = very good. This can reflect physiological health status more objectively and avoid measurement bias due to unobservable individual heterogeneity. The second is subjective self-rated health, which is a more credible indicator of physiological health widely used in social sciences (41), measured by the response to the question, “How do you rate your health at present?”. The third is subjective wellbeing, which measures mental health status from a broad perspective (42–44) through the response to the question “How do you rate your life at present?”. The subjective indicators were assigned a 5-point scale, ranging from 0 = very bad to 4 = very good.

### 4.3. Explanatory variables

According to the “Plan for the Construction of the Social Elderly Care Service System (2011–2015)” issued by the General Office of the State Council of China, community care services include aspects such as daily living assistance, rehabilitation care, medical care, psychological comfort, and legal services. We assessed community care services using the question “What kind of social services are available in your community?” with eight specific items in the questionnaire including aspects such as daily care services, health education, social psychological comfort, and legal services. The variable of community care services is a binary variable that is defined as equal to 1 if one or more services are available in the respondents' community and equal to 0 otherwise.

To further investigate the impact of the different types of community services, we classified eight specific items into four types and defined them in terms of binary variables. First, life care services such as “personal daily care services” and “daily shopping”; second, healthcare services such as “home visits” and “health education”; third, spiritual recreation services such as “psychological consulting” and “social and recreation activities”; fourth, legal advocacy services such as “human rights consulting services” and “neighboring relations”. The variables are defined as equal to 1 if one or more services are available in the respondents' community and equal to 0 otherwise.

### 4.4. Covariates

According to Grossman's (35) health production function, the health of older adults is also influenced by family support, income, activities of daily living (ADL), medical status, and lifestyle habits.

We used three indicators for family support as follows: living, financial, and emotional support. Living support is measured by the living pattern of the older adults, equal to 1 if living with adult children, and 0 otherwise. Financial support was measured through the survey question “How much money (including cash and value of materials) did you get last year from your children and their spouses whether they are living with you or not?”. This variable is defined as equal to 1 if the amount of support is ≥100 yuan and 0 otherwise. Third, emotional support was measured based on answers to the question “Who do you ask for help when you have problems or difficulties?” where a value of 1 was assigned if older adults think of their spouses, children, grandchildren, and their spouses first when they need help and 0 otherwise.

Income status includes pensions and primary means of financial support. First, the variable pensions are counted as 1 if the participants have many pensions (including commercial pension insurance). As there is a large proportion of older adults who do not have stable pensions or pensions that are sufficient to cover living expenses in China, the primary means of financial support are divided into three dummy variables as follows: financial independence = 1, if the primary means of financial support is through their pension or their spouse's pension; financial dependence = 1, if the primary means is through their child or other relatives; and government relief = 1, if the primary means is through government funding.

The binary variable ADL is defined as equal to 1 if there is no difficulty in performing ADL tasks (bathing, dressing, toileting, transferring, urine control, and eating) and equal to 0 if there are ADL limitations.

Medical status was measured using two variables: medical insurance and sick care. Medical insurance was defined as equal to 1 if the participant had any public or commercial medical insurance and 0 otherwise. Sick care was defined as 1 if family members provide care and 0 otherwise.

Health behavior was measured based on the smoking and drinking habits of the participants. Smoking and drinking were dummy variables coded as 1 if the respondent smoked or consumed alcohol. We also controlled for a range of demographic and socioeconomic factors, including age, residence, marital status, gender, education, and region.

### 4.5. Analysis

A linear regression model with a time-fixed effect was used to estimate the effect of community care services on the health of older adults.


(2) Hijt=β0+β1CSijt+φXijt'+θWt+γRj+μijt,

where *H*_*ijt*_ denotes the health of older adults *i* living in region *j* in year *t*. *CS*_*ijt*_ is the binary variable for whether the older adult's community has care services. $X_{ijt}^{{}^{\prime }}$ is a vector of covariates and control variables reflecting family support, medical and income status, ADL, health behavior, and demographic characteristics, which have proven to impact health in the previous Grossman model. *W*_*t*_ and *R*_*j*_ are full sets of year and region dummies, respectively, which account for the time period and regional fixed effects.

As the health status of older adults varies over time, and the health status and the subjective wellbeing of older adults may be influenced by regional factors, such as environmental conditions, socioeconomic factors, cultural norms, and healthcare resources, and these difficult-to-measure potential factors can affect our research findings. A linear regression model controlling for time periods and regional fixed effects allows us to estimate the effect of community care services on the health of older adults while accounting for potential confounding factors due to time periods and regions.

To further investigate the impact of different types of community services, we subdivided the community home care services into four types. The basic specification for the analysis is as follows:


(3) $$\begin{array}{l} H_{ijt}=\beta_{0}+\beta_{1}LC_{ijt}+\beta_{2}MS_{ijt}+\beta_{3}SR_{ijt}+\beta_{4}LA_{ijt}+\varphi X_{ijt}\text{'}\\ \;+\;\theta W_{t}+\gamma R_{j}+\mu_{ijt}, \end{array}$$

where *LC*_*ijt*_ is a community-based life care service; *MS*_*ijt*_ is a community-based healthcare service; *SR*_*ijt*_ is a community-based spiritual recreation service; and *LA*_*ijt*_ is a community-based legal advocacy service.

### 4.6. Robustness analysis

The relationship between community care services and the health of older adults is difficult to interpret because of omitted variables and the presence of a bidirectional link between the two. To avoid endogeneity bias, we first chose county-level community care services supply quantity (from 0 to 8) and four county-level supply rates (from 0 to 1) of life care, medical care, spiritual recreation, and legal advocacy services as instrumental variables (IVs) to verify the robustness of the results. However, it is difficult to search for IVs, since the data collected by CLHLS were randomly selected based on gender and place of residence. We use the information collected from the participants who live in the same county to calculate the county-level community care services supply quantity and the county-level supply rates of life care, medical care, spiritual recreation, and legal advocacy services. An IV analysis requires instruments that are highly correlated with endogenous variables and have no direct effect on the outcome (i.e., are uncorrelated with the error term). Only a handful of studies have used causal econometric methods to address reciprocal causation. The county-level community care service supply is cluster-level information, and the higher the county-level supply rate, the higher the probability that an individual is supplied and, therefore, has a strong correlation with whether an individual is supplied with community aged care services. At the same time, the supply of community care services at the county level cannot directly affect the individual health of people, thereby satisfying the exogenous condition of the instrumental variable. In this study, the F-statistic was evaluated, and over-identification tests were performed to ensure the validity of the IVs.

Second, an individual FE model was provided to further verify the robustness of the results. These unobserved confounding factors are generally considered stable across observation periods, and their effects come from unobservable factors that are offset by each other when examining the effects of key independent variables on the dependent variable, thus avoiding the influence of potentially unobservable confounding factors.

Third, as the key dependent variables in this study are in the order of connotation, we use an ordered logit model to further corroborate the parameter estimation.

## 5. Results

### 5.1. Descriptive statistics

Table 1 reports the summary statistics of the main variables. In the full sample, there were some measured differences between the objective and subjective variables. For example, 13.4% of older adults perceived their health to be poor or very poor, whereas CLHLS investigators observed that only 5.7% of older adults were unhealthy or very unhealthy.

**Table 1:** Summary statistics of main variables.

**Variable**	**Total**	**No service**	**With service**	**Age < 80**	**Age ≥ 80**	**Rural**	**Urban**	**Male**	**Female**
**Sample size**	**4,700**	**2,696**	**2,004**	**3,143**	**1,557**	**2,397**	**2,303**	**2,408**	**2,292**
**Explained variables**									
**Objective health**									
Very bad	0.005	0.004	0.005	0.005	0.004	0.006	0.003	0.005	0.004
Bad	0.052	0.050	0.054	0.047	0.061^**^	0.052	0.051	0.037	0.067^***^
Relative good	0.575	0.576	0.574	0.555	0.617^***^	0.576	0.574	0.548	0.604^***^
Very good	0.369	0.370	0.367	0.393	0.319^***^	0.365	0.372	0.409	0.325^***^
**Subjective health**									
Very bad	0.007	0.008	0.005	0.007	0.005	0.005	0.008	0.005	0.008
Bad	0.127	0.125	0.130	0.132	0.118	0.143	0.112^***^	0.110	0.146^***^
Common	0.359	0.354	0.366	0.355	0.368	0.347	0.372	0.360	0.359
Good	0.371	0.382	0.356^*^	0.372	0.369	0.377	0.365	0.373	0.369
Very good	0.136	0.131	0.143	0.134	0.140	0.128	0.143	0.152	0.118^***^
**Subjective wellbeing**									
Very bad	0.004	0.006	0.002^*^	0.005	0.003	0.004	0.004	0.002	0.006^*^
Bad	0.037	0.046	0.025^***^	0.042	0.028^**^	0.041	0.033	0.039	0.034
Common	0.344	0.362	0.320^***^	0.371	0.290^***^	0.372	0.316^***^	0.341	0.349
Good	0.438	0.436	0.440	0.422	0.469^***^	0.450	0.425	0.439	0.436
Very good	0.177	0.150	0.213^***^	0.160	0.210^***^	0.133	0.223^***^	0.178	0.175
**Explanatory variable**									
Community care services	0.426	0.000	1.000	0.396	0.487^***^	0.388	0.467^***^	0.431	0.421
Life care	0.074	0.000	0.173^***^	0.072	0.078	0.075	0.073	0.075	0.072
Medical care	0.303	0.000	0.711^***^	0.264	0.382^***^	0.257	0.351^***^	0.315	0.291^*^
Spiritual recreation	0.170	0.000	0.399^***^	0.158	0.193^***^	0.120	0.222^***^	0.177	0.162
Legal advocacy	0.258	0.000	0.605^***^	0.248	0.277^**^	0.242	0.274^***^	0.268	0.247
**Family support**									
Living support	0.309	0.298	0.323^*^	0.225	0.477^***^	0.277	0.342^***^	0.203	0.419^***^
Family financial support	0.856	0.852	0.862	0.859	0.850	0.896	0.815^***^	0.834	0.880^***^
Family emotional support	0.950	0.946	0.955	0.953	0.943	0.950	0.950	0.943	0.956^*^
**Medical status**									
Medical insurance	0.703	0.622	0.812^***^	0.662	0.786^***^	0.643	0.766^***^	0.737	0.668^***^
Sick care	0.717	0.793	0.614^***^	0.793	0.562^***^	0.780	0.651^***^	0.718	0.715
**Income status**									
Pension	0.348	0.300	0.412^***^	0.324	0.396^***^	0.177	0.525^***^	0.420	0.272^***^
Financial independence	0.468	0.473	0.462	0.523	0.357^***^	0.375	0.564^***^	0.576	0.354^***^
Financial dependence	0.440	0.455	0.421	0.396	0.531^***^	0.544	0.332^***^	0.333	0.553^***^
Government relief	0.092	0.073	0.117^***^	0.081	0.112^***^	0.081	0.103^***^	0.091	0.092
**Initial health**									
ADL	0.934	0.950	0.914^***^	0.963	0.877^***^	0.954	0.914^***^	0.946	0.923^***^
**Health behavior**									
Smoking	0.245	0.243	0.248	0.268	0.199^***^	0.277	0.211^***^	0.417	0.064^***^
Drinking	0.233	0.235	0.229	0.243	0.211^**^	0.267	0.197^***^	0.360	0.099^***^
**Demographic characteristics**									
Age	77.254	76.441	78.348^***^	73.072	85.697^***^	76.471	78.070^***^	77.642	76.848^***^
Gender (men = 0)	0.488	0.492	0.482	0.499	0.464^***^	0.495	0.480	0.000	1.000
Residence (rural = 0)	0.490	0.455	0.536^***^	0.463	0.545^***^	0.000	1.000	0.498	0.482
Education (illiterate = 0)	0.531	0.525	0.539	0.562	0.469^***^	0.460	0.605^***^	0.757	0.293^***^
Marriage (no spouse = 0)	0.559	0.570	0.542^*^	0.647	0.379^***^	0.575	0.541^**^	0.707	0.403^***^
**Region**									
Western Region	0.354	0.382	0.316^***^	0.343	0.377^**^	0.352	0.356	0.355	0.353
Central Region	0.272	0.274	0.270	0.271	0.274	0.290	0.254^***^	0.241	0.305^***^
Eastern Region	0.374	0.344	0.414^***^	0.386	0.349^**^	0.359	0.389^**^	0.404	0.342^***^
**Year of survey**									
2005	0.250	0.300	0.183^***^	0.315	0.118^***^	0.310	0.188^***^	0.250	0.250
2008	0.250	0.333	0.139^***^	0.281	0.187^***^	0.295	0.204^***^	0.250	0.250
2011	0.250	0.200	0.317^***^	0.232	0.286^***^	0.214	0.287^***^	0.250	0.250
2014	0.250	0.168	0.361^***^	0.171	0.408^***^	0.181	0.321^***^	0.250	0.250

^***^p < 0.01, ^**^p < 0.05, ^*^ *p* < 0.1.

We subgrouped the sample according to the supply of community care services, age, residence, and gender. The results showed that a significantly higher proportion of older adults with community care services were very satisfied with their lives (*p* < 0.01). A significantly lower proportion of older adults (age ≥ 80 years) had very good objective health (31.9 vs. 39.3%, *p* < 0.01), and a significantly higher proportion were very happy with their lives (21 vs. 16%, *p* < 0.01). Among the urban older adults, 22.3% were very satisfied with their lives, but this proportion was 13.3% in rural areas (*p* < 0.01). A significantly higher proportion of male older adults had very good objective health and a very happy life compared with female older adults (40.9 vs. 32.5%, *p* < 0.01; 15.2 vs. 11.8%, *p* < 0.01).

We list the community care service supply rates. The older adults responded with a total supply rate of 42.6% for community care services, and subtype supply rates of 7.4, 30.3, 17.0, and 25.8% for life care, medical care, spiritual recreation, and legal advocacy services, respectively. The proportion of rural residents with community care services was significantly lower than that of urban residents (38.8 vs. 46.7%, *p* < 0.01; 0.88 vs. 1.19, *p* < 0.01).

Among other explanatory variables, 30.9% of respondents lived with their children, 85.6% had family financial support, and 95% had emotional support from their families. Nearly half of the oldest older adults lived with their children, a proportion two times as high as that of older adults under 80 years (*p* < 0.01), implying that more care is needed with time. Rural seniors made up a lower proportion of those living with their children (*p* < 0.01) and a higher proportion of those living with their family financial support (*p* < 0.01). A lower proportion of men lived with their children and had financial or emotional support from their families (*p* < 0.01).

### 5.2. Estimation results for community care services and the health of older adults

Table 2 presents community care services as the independent variable. The estimation results using OLS models with time period and regional fixed effects show that community care services are associated with both objective health (evaluated by others) and the self-rated health and wellbeing of old people, therefore, providing a solid basis for further research. Comparing the impact coefficients, the promotion effect of wellbeing is more effective.

**Table 2:** OLS estimation results for community care services and the health of older adults.

	**(1)**	**(2)**	**(3)**
	**Objective**	**Subjective**	**Subjective**
**Variables**	**Health**	**Health**	**Wellbeing**
Community care services	0.035^*^ (0.021)	0.062^*^ (0.033)	0.109^***^ (0.030)
Family support	Yes	Yes	Yes
Medical status	Yes	Yes	Yes
Income status	Yes	Yes	Yes
Initial health	Yes	Yes	Yes
Health behavior	Yes	Yes	Yes
Demographic characteristics	Yes	Yes	Yes
Region FE	Yes	Yes	Yes
Year FE	Yes	Yes	Yes
Observations	4,700	4,700	4,700

^***^, ^**^, and ^*^ indicate significance level at 1%, 5%, and 10%, respectively. Robust standard errors with a cluster at the individual level are presented in parentheses.

To further investigate which type of service has a greater impact, Table 3 shows our preferred specification using OLS models with time period and regional fixed effects that assume community care services are exogenous and the IV models using county-level quantity and four types of supply rates as instruments for community care services. The results for the OLS models are reported in Panel A, with family support, medical status, income status, initial health, health behavior, and demographic category, controlling for a year- and region-fixed effects. In the OLS models, medical care services were significantly associated with a 0.079 increase in subjective wellbeing scores (*p* < 0.05). Spiritual recreation services were significantly associated with a 0.109 increase in objective health scores, a 0.135 increase in subjective health scores, and a 0.106 increase in subjective wellbeing scores (both *p* < 0.01).

**Table 3:** Estimation results for different types of community care services and the health of older adults.

	**Panel A: OLS**			**Panel B: IV**		
	**(1)**	**(2)**	**(3)**	**(4)**	**(5)**	**(6)**
	**Objective**	**Subjective**	**Subjective**	**Objective**	**Subjective**	**Subjective**
**Variables**	**Health**	**Health**	**Wellbeing**	**Health**	**Health**	**Wellbeing**
Life care services	0.044	0.012	0.039	0.028	−0.032	−0.034
	(0.036)	(0.055)	(0.047)	(0.048)	(0.077)	(0.068)
Medical care services	−0.034	−0.027	0.079^**^	−0.017	−0.005	0.123^***^
	(0.024)	(0.038)	(0.034)	(0.031)	(0.049)	(0.043)
Spiritual recreation services	0.109^***^	0.135^***^	0.106^***^	0.090^**^	0.117^*^	0.033
	(0.026)	(0.042)	(0.034)	(0.038)	(0.060)	(0.051)
Legal advocacy services	−0.014	−0.010	−0.027	−0.038	−0.017	−0.017
	(0.023)	(0.035)	(0.031)	(0.031)	(0.049)	(0.041)
Living support	−0.013	0.020	0.116^***^	−0.013	0.020	0.116^***^
	(0.027)	(0.045)	(0.039)	(0.025)	(0.041)	(0.035)
Family financial support	−0.001	−0.017	0.001	−0.000	−0.018	−0.000
	(0.026)	(0.039)	(0.036)	(0.025)	(0.039)	(0.034)
Family emotional support	0.096^**^	0.192^***^	0.202^***^	0.098^**^	0.192^***^	0.201^***^
	(0.045)	(0.069)	(0.066)	(0.044)	(0.066)	(0.064)
Medical insurance	−0.065^***^	−0.030	0.009	−0.064^***^	−0.030	0.010
	(0.023)	(0.037)	(0.032)	(0.022)	(0.035)	(0.031)
Sick care	0.025	−0.009	0.228^***^	0.025	−0.009	0.229^***^
	(0.047)	(0.082)	(0.074)	(0.047)	(0.082)	(0.076)
Pension	0.027	0.022	0.180^***^	0.028	0.024	0.185^***^
	(0.023)	(0.036)	(0.032)	(0.022)	(0.034)	(0.029)
Financial independence (Government relief = 0)	0.077^**^	0.117^**^	0.103^**^	0.077^**^	0.117^**^	0.104^**^
	(0.034)	(0.058)	(0.049)	(0.032)	(0.049)	(0.044)
Financial dependence (Government relief = 0)	0.004	0.054	0.122^**^	0.003	0.053	0.120^***^
	(0.032)	(0.056)	(0.047)	(0.031)	(0.050)	(0.044)
ADL	0.292^***^	0.248^***^	0.027	0.294^***^	0.249^***^	0.030
	(0.043)	(0.061)	(0.050)	(0.041)	(0.058)	(0.047)
Smoking	0.031	0.014	−0.038	0.031	0.014	−0.040
	(0.025)	(0.040)	(0.033)	(0.022)	(0.034)	(0.030)
Drinking	0.105^***^	0.183^***^	0.069^**^	0.105^***^	0.183^***^	0.068^**^
	(0.023)	(0.037)	(0.031)	(0.021)	(0.033)	(0.028)
Age	−0.003^*^	0.009^***^	0.010^***^	−0.003^**^	0.009^***^	0.010^***^
	(0.002)	(0.003)	(0.002)	(0.001)	(0.002)	(0.002)
Residence (rural = 0)	0.022	0.065^**^	0.059^**^	0.022	0.064^**^	0.061^**^
	(0.020)	(0.031)	(0.026)	(0.019)	(0.029)	(0.025)
Marriage (no spouse = 0)	−0.058^**^	−0.014	0.082^**^	−0.058^**^	−0.014	0.082^**^
	(0.027)	(0.047)	(0.041)	(0.024)	(0.040)	(0.035)
Gender (male = 0)	−0.054^**^	−0.053	0.057	−0.053^**^	−0.052	0.059^*^
	(0.027)	(0.044)	(0.037)	(0.022)	(0.035)	(0.031)
Education (illiterate = 0)	0.027	−0.040	0.048	0.027	−0.039	0.050^*^
	(0.023)	(0.039)	(0.033)	(0.019)	(0.031)	(0.027)
Region FE	Yes	Yes	Yes	Yes	Yes	Yes
Year FE	Yes	Yes	Yes	Yes	Yes	Yes
Observations	4,700	4,700	4,700	4,700	4,700	4,700
First stage F-statistic				801.223		
*P*-value if Hansen J-statistic				0.172	0.528	0.115

^***^, ^**^, and ^*^ indicate significance level at 1%, 5%, and 10%, respectively. Robust standard errors with a cluster at the individual level are presented in parentheses.

Panel B shows the results of the IV model after taking the county-level community care services supply quantity rate (from 0 to 8) and the life care, medical care, spiritual recreation, and legal advocacy service supply rates (from 0 to 1) as IV. The results show a significant positive correlation between community care services and the health of older adults. Medical care services led to an increase in subjective wellbeing scores; therefore, the coefficient increased from 0.079 to 0.123. Spiritual recreation services led to an increase of 0.090 in objective health scores (*p* < 0.05) and 0.117 in subjective health scores (*p* < 0.1).

The validity of an instrument relies on two conditions: high correlation with the endogenous variable and no direct effects on the outcome, conditional on the endogenous and exogenous variables. We examined the correlation between county-level IV and the four types of services, and the results show that the association is significantly positive, which is due to the higher county-level supply quantity and higher supply rates indicating a higher probability of an individual receiving a service. The values of the first-stage F-statistic 622.083 for IVs are much higher than the value of 10 for weak identification and the critical value of 26.87 for a 10% IV size, indicating that the chosen instruments are not weak. Next, we ran Hansen J's over-identifying test, given the presence of multiple instruments. The tests were not significant (*p* > 0.05), and the Hansen J-statistic failed to reject the null hypothesis of exogenous instruments.

The regression results for other explanatory variables were more consistent in the OLS and IV models and generally consistent with the theoretical model setting. Regarding family support, living with children and emotional support significantly enhanced the health and wellbeing of older adults. We found that the health gain from family support was stronger than that of community care services when comparing the impact coefficients. Thus, community care provides the necessary support for the overall policy direction of home care. For medical care, sick care is more effective than health insurance in enhancing the health of older adults. With regard to income, having a pension significantly enhanced the wellbeing of older adults. Furthermore, ADL affects the health of older adults and smoking had no significant effect on the estimation of health behaviors, whereas drinking significantly improved health and life satisfaction. In addition, according to the regression results, men were healthier than women, and the wellbeing indices were significantly lower in rural compared to urban areas, which is generally consistent with the descriptive statistics (Table 1). The results of the IV model show that the objective health scores decrease significantly as age increases, but subjective health and wellbeing in life increase. The analysis of descriptive statistics (Table 1) also shows that the health status of people over 80 years is worse but for the wellbeing index, this is higher.

In summary, the OLS and IV estimates are generally consistent, which suggests that our empirical findings are relatively robust to the specifications that account for other potentially confounding variables.

### 5.3. Robustness checks

Our preferred specification, which controls for a time period and regional fixed effects, shows that the two types of community care services are significantly associated with the health of older adults. We found IVs and the results supported the robustness of the OLS model results. However, the consistency of the estimated quantities relies on the exogeneity of the instruments that are difficult to validate. The results of the individual FE models are provided in Table 4 to further verify the robustness of the results. The individual FE models could eliminate unobserved heterogeneity using the estimator in the panel data regression and require no exclusion restrictions for the identification of instruments (45). Table 4 Panel A presents the results from individual FE regression analyses with control variables, where medical care and spiritual recreation services had a positive effect on the health of older adults, and the results for the other explanatory variables were consistent with the OLS and IV models.

**Table 4:** Robustness checks.

	**Panel A: Individual FE**			**Panel B: Ordered Logit**		
	**(1)**	**(2)**	**(3)**	**(4)**	**(5)**	**(6)**
	**Objective**	**Subjective**	**Subjective**	**Objective**	**Subjective**	**Subjective**
**Variables**	**Health**	**Health**	**Wellbeing**	**Health**	**Health**	**Wellbeing**
Life care services	0.037	0.000	−0.003	0.206	0.016	0.073
	(0.039)	(0.055)	(0.050)	(0.139)	(0.123)	(0.128)
Medical care services	−0.009	0.015	0.060^*^	−0.111	−0.030	0.197^**^
	(0.026)	(0.038)	(0.035)	(0.096)	(0.086)	(0.090)
Spiritual recreation services	0.054^*^	0.056	0.072^*^	0.353^***^	0.254^***^	0.234^**^
	(0.028)	(0.044)	(0.039)	(0.102)	(0.096)	(0.092)
Legal advocacy services	0.001	0.033	0.033	−0.040	0.013	−0.008
	(0.025)	(0.036)	(0.033)	(0.091)	(0.082)	(0.082)
Family support	Yes	Yes	Yes	Yes	Yes	Yes
Medical status	Yes	Yes	Yes	Yes	Yes	Yes
Income status	Yes	Yes	Yes	Yes	Yes	Yes
Initial health	Yes	Yes	Yes	Yes	Yes	Yes
Health behavior	Yes	Yes	Yes	Yes	Yes	Yes
Demographic characteristics	Yes	Yes	Yes	Yes	Yes	Yes
Region FE	Yes	Yes	Yes	Yes	Yes	Yes
Year FE	Yes	Yes	Yes	Yes	Yes	Yes
Individual FE	Yes	Yes	Yes			
Observations	4,700	4,700	4,700	4,700	4,700	4,700

^***^, ^**^, and ^*^ indicate significance level at 1%, 5%, and 10%, respectively. Robust standard errors with a cluster at the individual level are presented in parentheses.

As the variable values of objective health, subjective health, and subjective wellbeing in this study are in order of connotation, Panel B in Table 4 reports the results under the use of an ordered logit model to further corroborate the robustness and credibility of the parameter estimation results.

### 5.4. Marginal effects

To explain the effects of different types of services more directly and specifically, Table 5 supplements the calculation of the marginal effects of the four types of services on the objective and subjective health and subjective wellbeing of older adults.

**Table 5:** Marginal effects of community care services on the health of older adults.

	**Very bad**	**Bad**	**Relative good**	**Good**	
**Panel A: objective health**					
Life care services	−0.001	−0.009	−0.030	0.040	
	(0.001)	(0.006)	(0.020)	(0.027)	
Medical care services	0.001	0.005	0.016	−0.021	
	(0.000)	(0.004)	(0.014)	(0.019)	
Spiritual recreation services	−0.002^***^	−0.016^***^	−0.051^***^	0.068^***^	
	(0.001)	(0.005)	(0.015)	(0.020)	
Legal advocacy services	0.000	0.002	0.006	−0.008	
	(0.000)	(0.004)	(0.013)	(0.017)	
**Panel B: subjective HEALTH**					
	**Very bad**	**Bad**	**Common**	**Good**	**Very good**
Life care services	−0.000	−0.001	−0.002	0.002	0.002
	(0.001)	(0.012)	(0.012)	(0.012)	(0.013)
Medical care services	0.000	0.003	0.003	−0.003	−0.003
	(0.001)	(0.008)	(0.009)	(0.009)	(0.009)
Spiritual recreation services	−0.002^**^	−0.024^***^	−0.026^***^	0.026^***^	0.026^***^
	(0.001)	(0.009)	(0.010)	(0.010)	(0.010)
Legal advocacy services	−0.000	−0.001	−0.001	0.001	0.001
	(0.001)	(0.008)	(0.008)	(0.008)	(0.008)
**Panel C: subjective wellbeing**					
Life care services	−0.000	−0.002	−0.011	0.005	0.009
	(0.001)	(0.004)	(0.020)	(0.009)	(0.016)
Medical care services	−0.001^*^	−0.007^**^	−0.031^**^	0.014^**^	0.025^**^
	(0.000)	(0.003)	(0.014)	(0.006)	(0.011)
Spiritual recreation services	−0.001^**^	−0.008^**^	−0.037^**^	0.016^**^	0.029^**^
	(0.000)	(0.003)	(0.015)	(0.007)	(0.012)
Legal advocacy services	0.000	0.000	0.001	−0.001	−0.001
	(0.000)	(0.003)	(0.013)	(0.006)	(0.010)

^***^, ^**^, and ^*^ indicate significance level at 1%, 5%, and 10%, respectively. Robust standard errors are presented in parentheses.Control variables include family support characteristics, medical status characteristics, income status characteristics, ADL, health behavior characteristics, demographic characteristics, and year- and region-fixed effects.

From the objective health section of Panel A in Table 5, the probability of having “very good” objective health increased by 6.8% and the probability of having “relative health,” “bad,” and “very bad” health decreased by 5.1, 1.6, and 0.2%, respectively, compared to the reference group that received no community care services. When comparing the impact coefficients, the effect of spiritual recreation services on older adults with poor health was smaller, but they were higher for older adults with good health.

The subjective health section of Panel B showed that, compared to the reference group without spiritual recreation services, the probability of having “very bad”, “bad”, and “common” subjective health decreased by 0.2, 2.4 and 2.6%; the probability of having “good”, and “very good” subjective health increased by 2.6 and 2.6%. This shows that, in general agreement with the findings of the objective indicators, the transition from absence to availability of spiritual recreation services has a more average effect on all but the worst health conditions of older adults.

The subjective wellbeing component of Panel C shows that all other factors being equal, the availability of healthcare and spiritual recreation service provision makes older adults 2.5 and 2.9% more likely to feel very happy, while reducing the probability of feeling unhappy and very unhappy, respectively, compared to a reference group with no community care services.

Community-provided home care services have less impact on the older adults with the poorest health scores and require more specialized therapeutic or rehabilitative care for those with very poor health.

### 5.5. Heterogeneity analysis

There may be urban–rural gender and age differences in the impact of community-based care services on health. Tables 6–8 present the heterogeneity analyses to determine this.

**Table 6:** Heterogeneous effect by residence.

	**Rural**			**Urban**		
	**(1)**	**(2)**	**(3)**	**(4)**	**(5)**	**(6)**
	**Objective**	**Subjective**	**Subjective**	**Objective**	**Subjective**	**Subjective**
**Variables**	**Health**	**Health**	**Wellbeing**	**Health**	**Health**	**Wellbeing**
**Panel A: OLS**						
Life care services	0.055	0.020	0.058	0.044	0.005	0.031
	(0.051)	(0.078)	(0.064)	(0.048)	(0.080)	(0.072)
Medical care services	−0.100	−0.023	0.107^**^	0.019	−0.037	0.050
	(0.035)	(0.055)	(0.047)	(0.032)	(0.052)	(0.048)
Spiritual recreation services	0.130^***^	0.122^*^	0.083^*^	0.074^**^	0.148^***^	0.128^***^
	(0.040)	(0.064)	(0.048)	(0.033)	(0.055)	(0.048)
Legal advocacy services	−0.040	−0.043	−0.050	0.013	0.012	−0.009
	(0.031)	(0.049)	(0.042)	(0.033)	(0.050)	(0.044)
**Panel B: IV**						
Life care services	0.035	−0.001	−0.032	0.034	−0.075	−0.035
	(0.070)	(0.108)	(0.088)	(0.065)	(0.113)	(0.106)
Medical care services	0.094^**^	−0.007	0.153^**^	0.046	−0.018	0.087
	(0.047)	(0.073)	(0.060)	(0.041)	(0.067)	(0.062)
Spiritual recreation services	0.138^**^	0.091	−0.055	0.011	0.130	0.085
	(0.059)	(0.093)	(0.071)	(0.049)	(0.080)	(0.074)
Legal advocacy services	0.084^*^	−0.037	−0.016	0.015	−0.011	−0.027
	(0.044)	(0.071)	(0.055)	(0.043)	(0.067)	(0.061)
First stage *F*-statistic	367.392			404.672		
*P*-value if Hansen *J*-statistic	0.2650	0.8455	0.1418	0.4448	0.2312	0.2794

^***^, ^**^, and ^*^ indicate significance level at 1%, 5%, and 10%, respectively. Robust standard errors are presented in parentheses. Control variables include family support characteristics, medical status characteristics, income status characteristics, ADL, health behavior characteristics, demographic characteristics, and year- and region-fixed effects.

**Table 7:** Heterogeneous effect by age.

	**65**<**Age**<**80**			**Age** ≥**80**		
	**(1)**	**(2)**	**(3)**	**(4)**	**(5)**	**(6)**
	**Objective**	**Subjective**	**Subjective**	**Objective**	**Subjective**	**Subjective**
**Variables**	**Health**	**Health**	**Wellbeing**	**Health**	**Health**	**Wellbeing**
**Panel A: OLS**						
Life care services	0.040	−0.041	0.045	0.057	0.110	0.041
	(0.044)	(0.067)	(0.062)	(0.064)	(0.091)	(0.068)
Medical care services	−0.070	−0.055	0.065	0.033	0.029	0.103^**^
	(0.031)	(0.045)	(0.042)	(0.037)	(0.062)	(0.051)
Spiritual recreation services	0.136^***^	0.136^*^	0.147^***^	0.050	0.120^*^	0.033
	(0.031)	(0.052)	(0.043)	(0.045)	(0.070)	(0.053)
Legal advocacy services	−0.003	0.003	−0.031	−0.032	−0.024	−0.015
	(0.029)	(0.043)	(0.040)	(0.035)	(0.058)	(0.047)
**Panel B: IV**						
Life care services	0.049	−0.117	−0.021	0.010	0.144	−0.028
	(0.058)	(0.094)	(0.087)	(0.089)	(0.138)	(0.109)
Medical care services	−0.053	−0.035	0.120^**^	0.048	0.066	0.124^*^
	(0.040)	(0.062)	(0.057)	(0.050)	(0.080)	(0.067)
Spiritual recreation services	0.115^**^	0.069	0.038	0.032	0.175^*^	0.011
	(0.047)	(0.075)	(0.066)	(0.064)	(0.099)	(0.080)
Legal advocacy services	−0.051	0.017	−0.048	−0.015	−0.070	0.042
	(0.040)	(0.061)	(0.052)	(0.050)	(0.083)	(0.067)
First stage *F*-statistic	524.59			236.798		
*P*-value if Hansen *J*-statistic	0.764	0.410	0.053	0.034	0.8637	0.8868

^***^, ^**^ and ^*^ indicate significance level at 1%, 5%, and 10%, respectively. Robust standard errors are presented in parentheses. Control variables include family support characteristics, medical status characteristics, income status characteristics, ADL, health behavior characteristics, demographic characteristics, and year- and region-fixed effects.

**Table 8:** Heterogeneous effect by gender.

	**Female**			**Male**		
	**(1)**	**(2)**	**(3)**	**(4)**	**(5)**	**(6)**
	**Objective**	**Subjective**	**Subjective**	**Objective**	**Subjective**	**Subjective**
**Variables**	**Health**	**Health**	**Wellbeing**	**Health**	**Health**	**Wellbeing**
**Panel A: OLS**						
Life care services	0.017	−0.108	−0.008	0.062	0.121^*^	−0.023
	(0.055)	(0.085)	(0.069)	(0.046)	(0.070)	(0.090)
Medical care services	−0.073	−0.023	0.086^*^	0.002	−0.027	0.070
	(0.035)	(0.054)	(0.050)	(0.034)	(0.053)	(0.057)
Spiritual recreation services	0.123^***^	0.195^***^	0.131^***^	0.090^**^	0.081	0.120^**^
	(0.039)	(0.061)	(0.047)	(0.035)	(0.058)	(0.061)
Legal advocacy services	−0.021	−0.027	−0.081	−0.008	0.005	0.020
	(0.033)	(0.052)	(0.044)	(0.032)	(0.047)	(0.056)
**Panel B: IV**						
Life care services	0.023	−0.095	0.011	0.022	0.045	−0.079
	(0.069)	(0.112)	(0.094)	(0.068)	(0.107)	(0.100)
Medical care services	−0.103	0.040	0.181^***^	0.065	−0.063	0.058
	(0.043)	(0.069)	(0.061)	(0.045)	(0.070)	(0.061)
Spiritual recreation services	0.114^**^	0.082	0.047	0.039	0.123	−0.020
	(0.053)	(0.083)	(0.073)	(0.053)	(0.087)	(0.072)
Legal advocacy services	−0.042	−0.070	−0.147^*^	−0.036	0.044	0.118^**^
	(0.044)	(0.068)	(0.056)	(0.043)	(0.070)	(0.059)
First stage *F*-statistic	403.073			367.115		
*P-*value if Hansen *J*-statistic	0.8289	0.8269	0.2598	0.1013	0.1113	0.1730

^***^, ^**^, and ^*^ indicate significance level at 1%, 5%, and 10%, respectively. Robust standard errors are presented in parentheses. Control variables include family support characteristics, medical status characteristics, income status characteristics, ADL, health behavior characteristics, demographic characteristics, and year- and region-fixed effects.

According to the OLS and IV regression results for rural residents, community-provided medical care services significantly improved wellbeing for the rural older adults but were not significant for the urban older adults. Spiritual recreation services significantly improved the objective health of rural older adults, whereas the effect on urban older adults was not significant under IV models. Since it is more inconvenient for rural older adults to visit a doctor, they worry about the high cost of medical care and do not go to the doctor because they are worried about care after illness. The health support effect on rural older adults may be more effective if the community or village can provide treatment and healthcare for common and chronic diseases and arrange health training seminars (46, 47).

Estimates stratified by age groups reported that community services do not affect the objective health of the oldest people, but significantly improve their subjective health and wellbeing. For those under 80 years of age, the gain effect of spiritual recreation services on objective health was more stable across the models.

For the female older adults, the enhancement effect of medical care services on female residents' wellbeing and the enhancement effect of spiritual recreation services on their objective health were stable under OLS and IV models. For the male older adults, the enhancement effect of spiritual recreation services on objective health and wellbeing in life under OLS models was no longer significant under the IV regression. Legal advocacy services had an enhancing effect on male residents' wellbeing in IV models.

In summary, spiritual recreation services had a general advantage on the health of all older adult groups, while medical care services significantly increased the subjective wellbeing of the oldest female older adults living in rural areas, and legal advocacy services could have improved the wellbeing of male older adults.

## 6. Discussion

Rapid population aging has led China to consider the introduction of community care services as an essential component of a comprehensive social care package. Using nationally representative survey data from China, this study investigates the impact of community care services on the health of older adults. The results from the OLS and IV models show that community care services led to significant health enhancement for older adults. The impact effects are most pronounced in spiritual recreation services and medical care. Further evidence suggests that the health-enhancement effect of spiritual recreation services is more effective for rural areas, men, and older adults under 80 years and that the health-enhancement effect of medical care services is more effective for rural areas, women, and older adults over 80 years. The effect of community care services was not significant for older adults with the poorest health scores but could significantly improve their quality of life and opportunities to enjoy a sense of wellbeing. Our findings extend the previous research on the relationship between community care services and health in developing countries.

The Chinese government has considered community care as a means of maintaining older adults' independence in their homes for as long as possible, and its current aging policy provides a wide range of services to support older adults in terms of extending active aging and improving their quality of life. However, our findings found that, among the various services currently provided in the community, life care-type services did not have a significant effect on the health and wellbeing of older adults. Sociocultural norms may help explain this finding, as older adults often expect adult children to provide for them, which is consistent with the literature that presents a problem-based perspective. The supply rate of life care services is too low, and the current capacity, professionalism, and quality of services do not meet the care needs of older adults (48, 49). Therefore, community care providers need knowledge and skills in physical, psychological, and medical care. Furthermore, policies and the availability of social resources, especially in conjunction with the development of long-term care insurance, are necessary to provide targeted services to help older adults improve their health and wellbeing, which is a meaningful research direction for the future.

Based on the above research findings, we recommend policymakers to take the following targeted policy measures: First, the supply of community-based medical care services and spiritual recreation services should be strengthened, especially in rural areas. This can be achieved by providing more resources and support to primary care organizations or community care centers in rural areas, and by encouraging the organization of spiritual recreation activities that are popular among older adults. Second, the healthcare needs of older women and those over 80 years should be focused on and more convenient and considerate medical care services should be provided. Meanwhile, for older men and those under 80 years, the supply of spiritual recreation services should be enhanced, and more attractive and diverse community entertainment activities should be provided. Finally, more attention should be given to older adults with poor health conditions, more community care services should be provided, and their quality of life and sense of wellbeing should be improved. These policy measures can improve the health of older adults while narrowing the urban–rural health gap and promoting the healthy development of aging.

The study has several limitations. First, we use three outcome variables to measure the health of older adults, the subjective self-rated health and subjective wellbeing indicators are measures that rely on the self-perception of the participants, which may be influenced by subjective biases and cultural factors. Although we used objectively observed health conditions by medical students as one of the outcomes, these measures may not fully capture the complexity of health status and may not be sufficient to provide a comprehensive assessment of the health of older adults. The potential implications of these biases and factors need future research. Second, due to data limitations, we used the availability of four types of community care services in the older adults' residing communities as the explanatory variables. In the future, further research should be conducted on more types of community care services, their quality, pricing, and usage, focusing on exploring the mechanisms behind the observed impact of community care services on health and identifying the most effective types of community care services for specific subgroups of older adults. Finally, it should be noted that there is the possibility of an endogeneity bias in this research. Finding instrumental variables is difficult. Although we used county-level community care services as instrumental variables, it assumes all older adults living within the same county have equal access to and utilize the same community care services, which may not be the case. Thus, we developed the individual FE models and the ordered logit model to further corroborate the robustness and credibility of the parameter estimation results. While this robustness check does not allow us to completely reject the hypothesis of an endogenous relationship, our results should, therefore, be interpreted with caution and seen as an entry point for more detailed investigation.

## Data availability statement

Publicly available datasets were analyzed in this study. This data can be found here: The data underlying the results presented in the study are available from the Chinese Longitudinal Healthy Longevity Survey (CLHLS), organized by Peking University. Anyone can access through application with the Peking University Open Research Data Platform (https://opendata.pku.edu.cn).

## Ethics statement

The studies involving human participants were reviewed and approved by Peking University. The patients/participants provided their written informed consent to participate in this study.

## Author contributions

WM: conceptualization and validation. WM and ZS: methodology, formal analyses, investigation, writing—original draft preparation, and writing—reviewing and editing. Both authors contributed to the article and approved the submitted version.

## Footnotes

^1^Source of data: National Health and Welfare Commission, Department of Aging and Health, “National Aging Development Bulletin 2021” http://www.gov.cn/xinwen/2022-10/26/content_5721786.htm.

## References

[1] Nieboer P Cramm JM. Age-friendly communities matter for older people's well-being. J Happiness Stud. (2018) 19:2405–20. 10.1007/s10902-017-9923-5

[2] Zhou J Walker A. The impact of community care services on the preference for ageing in place in urban China. Health Soc Care Community. (2021) 29:1041–50. 10.1111/hsc.13138 32783285

[3] Laporte A Croxford R Coyte P C. Can a publicly funded home care system successfully allocate service based on perceived need rather than socioeconomic status? A Canadian experience. Health Soc Care Commun. (2007) 15:108–19. 10.1111/j.1365-2524.2006.00672.x 17286672

[4] Blomgren J Martikainen P Martelin T Koskinen S. Determinants of home-based formal help in community-dwelling older people in Finland. Eur J Ageing. (2008) 5:335–47. 10.1007/s10433-008-0094-4 28798584 PMC5546292

[5] Chang F Shi Y Yi H Johnson N. Adult child migration and elderly parental health in rural China. China Agric Econ Rev. (2016) 8:677–97. 10.1108/CAER-11-2015-0169

[6] Baozhong S Yuheng L Xiaodong Z. Who are to support the aged in rural China? The study of people's willingness to purchase socialized care service and its influencing factors. J Rural Stud. (2022) 93:496–503. 10.1016/j.jrurstud.2019.12.017

[7] Li T Yu W Baležentis T Zhu J Ji Y. Rural demographic change, rising wages and the restructuring of Chinese agriculture. China Agric Econ Rev. (2017) 9:478–503. 10.1108/CAER-02-2016-0025

[8] Feng Z Liu C Guan X Mor V. China's rapidly aging population creates policy challenges in shaping a viable long-term care system. Health Aff. (2012) 31:2764–73. 10.1377/hlthaff.2012.0535 23213161 PMC3711106

[9] Chen YJ. Strength perspective: an analysis of ageing in place care model in Taiwan based on traditional filial piety. Ageing Int. (2008) 32:183–204. 10.1007/s12126-008-9018-z

[10] General General Office of the State Council PRC. Development of National Aging Career and Senior Care Service System During the 14th Five Year Plan. (2011). Retrieved from: https://www.mca.gov.cn/article/xw/mtbd/202202/20220200039833.shtml (accessed February 22, 2022).

[11] Birren JE Dieckmann L. Concepts and Content of Quality of Life in the Later Years: An Overview. Elsevier Science. (1991) 17:344–60.

[12] Zunzunegui MV Beland F Otero A. Support from children, living arrangements, self-rated health and depressive symptoms of older people in Spain. Int J Epidemiol. (2001) 30:1090–9. 10.1093/ije/30.5.1090 11689528

[13] Gu D Dupre ME Liu G. Characteristics of the institutionalized and community-residing oldest-old in China. Soc Sci Med. (2007) 64:871–83. 10.1016/j.socscimed.2006.10.026 17126971

[14] Lund R Due P Modvig J Holstein BE Damsgaard MT Andersen PK. Cohabitation and marital status as predictors of mortality—an eight year follow-up study. Soc Sci Med. (2002) 55:673–9. 10.1016/S0277-9536(01)00219-2 12188471

[15] Liu H Gao S Wang J. The impact of elder-care patterns on Chinese elderly's health and well-being. Econ Res J. (2011) 4:80–93 (in Chinese).

[16] Caron CD Ducharme F Griffith J. Deciding on institutionalization for a relative with dementia: the most difficult decision for caregivers. Can J Aging. (2006) 25:193–205. 10.1353/cja.2006.0033 16821201

[17] Légaré F Brière N Stacey D Bourassa H Desroches S Dumont S. improving decision making on location of care with the frail elderly and their caregivers (the DOLCE study): study protocol for a cluster randomized controlled trial. Trials. (2015) 16:1–8. 10.1186/s13063-015-0567-7 25881122 PMC4337186

[18] Boland L Légaré F Perez MMB Menear M Garvelink MM McIsaac DI. Impact of home care versus alternative locations of care on elder health outcomes: an overview of systematic reviews. BMC Geriatr. (2017) 17:1–15. 10.1186/s12877-016-0395-y 28088166 PMC5237488

[19] World Health Organization. Global Strategy and Action Plan on Ageing and Health (2017). p. 15–16.

[20] Wiles JL Leibing A Guberman N Reeve J Allen RE. The meaning of “aging in place” to older people. Gerontologist. (2012) 52:357–66. 10.1093/geront/gnr098 21983126

[21] Mah JC Stevens SJ Keefe JM Rockwood K Andrew MK. Social factors influencing utilization of home care in community-dwelling older adults: a scoping review. BMC Geriatr. (2021) 21:1–21. 10.1186/s12877-021-02069-1 33639856 PMC7912889

[22] Brach JS VanSwearingen JM. Physical impairment and disability: relationship to performance of activities of daily living in community-dwelling older men. Phys Ther. (2002) 82:752–61. 10.1093/ptj/82.8.752 12147005

[23] Hellström Y Persson G Hallberg IR. Quality of life and symptoms among older people living at home. J Adv Nurs. (2004) 48:584–93. 10.1111/j.1365-2648.2004.03247.x 15548249

[24] King AII Parsons M Robinson E Jörgensen D. Assessing the impact of a restorative home care service in New Zealand: a cluster randomised controlled trial. Health Soc Care Commun. (2012) 20:365–74. 10.1111/j.1365-2524.2011.01039.x 22106952

[25] Stuck AE Aronow HU Steiner A Alessi CA Büla CJ Gold MN. A trial of annual in-home comprehensive geriatric assessments for elderly people living in the community. N Engl J Med. (1995) 333:1184–9. 10.1056/NEJM199511023331805 7565974

[26] Huss A Stuck AE Rubenstein LZ Egger M Clough-Gorr KM. Multidimensional preventive home visit programs for community-dwelling older adults: a systematic review and meta-analysis of randomized controlled trials. J Gerontol Ser A Biol Sci Med Sci. (2008) 63:298–307. 10.1093/gerona/63.3.298 18375879

[27] You E Dunt DR Doyle C. Case managed community aged care: what is the evidence for effects on service use and costs? J Aging Health. (2013) 25:1204–42. 10.1177/0898264313499931 23958520

[28] Chapman SA Keating N Eales J. Client-centred, community-based care for frail seniors. Health Soc Care Commun. (2003) 11:253–61. 10.1046/j.1365-2524.2003.00420.x 12823430

[29] Gaugler JE Jarrott SE Zarit SH Stephens MA Townsend A Greene R. Adult day service use and reductions in caregiving hours: effects on stress and psychological well-being for dementia caregivers. Int J Geriatr Psychiatry. (2003) 18:55–62. 10.1002/gps.772 12497556

[30] Yang L Wang L Di X Dai X. Utilisation of community care services and self-rated health among elderly population in China: a survey-based analysis with propensity score matching method. BMC Public Health. (2021) 21:1–11. 10.1186/s12889-021-11989-x 34696767 PMC8546940

[31] Lv X Zhang X. The influence of community home-based elderly care on the health of the elderly population. Chin J Popul Sci. (2022) 3:111–25.

[32] Gu T Li D Li L. The elderly's demand for community-based care services and its determinants: a comparison of the elderly in the affordable housing community and commercial housing community of China. J Healthc Eng. (2020) 2020:13–26. 10.1155/2020/1840543 33144931 PMC7596538

[33] Cheng X. Is the community-based elderly care service effective —Empirical test based on the life quality of the elderly. Soc Sec Stud. (2019) 3:8–25. 10.3969/j.issn.1674-4802.2019.03.003 (in Chinese).

[34] Wong YC Leung J. Long-term care in China: issues and prospects. J Gerontol Soc Work. (2012) 55:570–86. 10.1080/01634372.2011.650319 22963116

[35] Grossman M. On the concept of health capital and the demand for health. J Polit Econ. (1972) 80:223–55. 10.1086/259880

[36] Bolin K Lindgren B Lindström M Nystedt P. Investments in social capital—implications of social interactions for the production of health. Soc Sci Med. (2003) 56:2379–90. 10.1016/S0277-9536(02)00242-3 12742602

[37] Galama TJ Van Kippersluis H. Health inequalities through the lens of health-capital theory: issues, solutions, and future directions. Health Inequality. (2013) 21:263–84. 10.1108/S1049-2585(2013)0000021013 24570580 PMC3932058

[38] Jacobson L. The family as producer of health—an extended Grossman model. J Health Econ. (2000) 19:611–37. 10.1016/S0167-6296(99)00041-7 11184796

[39] Huang C Elo IT. Mortality of the oldest old Chinese: the role of early-life nutritional status, socio-economic conditions, and sibling sex-composition. Popul Stud. (2009) 63:7–20. 10.1080/00324720802626921 19184718 PMC2832602

[40] Yi Z. Introduction to the chinese longitudinal healthy longevity survey (CLHLS). Healthy Long China. (2008) 20:23–38. 10.1007/978-1-4020-6752-5_2

[41] Jylhä M. What is self-rated health and why does it predict mortality? Towards a unified conceptual model. Soc Sci Med. (2009) 69:307–16. 10.1016/j.socscimed.2009.05.013 19520474

[42] Marzo RR Khanal P Ahmad A Rathore FA Chauhan S Singh A. Quality of life of the elderly during the COVID-19 pandemic in Asian countries: a cross-sectional study across six countries. Life. (2022) 12:365. 10.3390/life12030365 35330116 PMC8948612

[43] Netuveli G Wiggins RD Hildon Z Montgomery SM Blane D. Quality of life at older ages: evidence from the English longitudinal study of aging (wave 1). J Epidemiol Commun Health. (2006) 60:357–63. 10.1136/jech.2005.040071 16537355 PMC2566174

[44] Chen F Short SE. Household context and subjective well-being among the oldest old in China. J Fam Issues. (2008) 29:1379–403. 10.1177/0192513X07313602 19554216 PMC2701306

[45] Ates M. Does grandchild care influence grandparents' self-rated health? Evidence from a fixed effects approach. Soc Sci Med. (2017) 190:67–74. 10.1016/j.socscimed.2017.08.021 28843872

[46] Beswick AD Gooberman-Hill R Smith A Wylde V Ebrahim S. Maintaining independence in older people. Rev Clin Gerontol. (2010) 20:128–53. 10.1017/S0959259810000079

[47] Fens M Vluggen T van Haastregt JCM Verbunt JA Beusmans GH van Heugten CM. Multidisciplinary care for stroke patients living in the community: a systematic review. J Rehabil Med. (2013) 45:321–30. 10.2340/16501977-1128 23546307

[48] Ding ZH Wang LL. Study on the equalization of home-based care services for the elderly in China. Popul J. (2011) 5:83–8 (in Chinese).

[49] Zhou J Walker A. The need for community care among older people in China. Ageing Soc. (2016) 36:1312–32. 10.1017/S0144686X1500034332517576

